# A Refresher of the Original Bloch’s Law Paper (Bloch, July 1885)

**DOI:** 10.1177/2041669515593043

**Published:** 2015-08-21

**Authors:** Andrei Gorea

**Affiliations:** Laboratoire Psychologie de la Perception, Université Paris Descartes and CNRS, Paris, France

**Keywords:** detection, temporal integration, information accumulation, linear systems, transfer function, drift-diffusion

## Abstract

In 1885, Adolphe-Moïse Bloch asked the following simple question “Is there a law describing the relationship between the duration of a light and its perceived intensity?” Based on a series of experiments using a *Foucault regulator* and a candle, Bloch concluded that “when the lighting duration varies from 0.00173 to 0.0518 seconds (…) *the [visible] light is markedly in inverse proportion to its duration*”—his famous law. As this law pertains to the more general and hotly debated question of accumulation of sensory information over time, it is timely to offer the public a full translation of Bloch’s original paper (from French) and to present it within the context of contemporary research.

## Introduction

### The Topic

Bloch’s Law (Bloch, 1885) states that, for relatively short presentations, the product of luminance (or contrast) and duration is constant at the detection threshold. Adherence to Bloch’s Law is progressively lost as stimulus duration increases. In the remainder, I refer to empirically derived threshold intensity versus duration functions as Bloch’s curves. Essentially, Bloch’s curves describe how the visual system acquires information over time in detection tasks.

### Some History

The first linear systems approach to visual perception (inspired from Ohm’s law on the decomposition of arbitrary periodic sounds into sinusoidal partials—see Heller, 2013) was introduced by De Lange (1958) and further developed by Kelly (1961, 1966, 1971) and Robson (1966). Fourier analysis pervades thereafter research in spatial vision (Campbell & Robson, 1968).

In 1972, Roufs starts a series of studies meant to establish the relation between temporal and temporal frequency visual sensitivity (Roufs, 1972a,b) and eventually suggests a method of deriving the temporal impulse response of the visual system (Roufs & Blommaert, 1981).

The direct link between sensitivity to temporal frequency (the *transfer function*) and to stimulus duration (Bloch’s curve) via the temporal impulse response of the visual system is further exposed by Koenderink and van Doorn (1978) and by Watson (1979, 1981, 1982, 1986) who proposes a simplified modeling of Bloch’s curve within the framework of high threshold theory.

As pointed out by Gorea and Tyler (1986, 2013), Watson’s mathematical formulation of Bloch’s curve fails to account for at least two of Bloch’s curve empirical characteristics, namely its nonmonotonicity (for low-spatial frequencies) and its progressively shallower slope with duration (for high-spatial frequencies). To account for those, Gorea and Tyler (1986) combined a high threshold and a signal detection approach involving multiple and independent nonlinear signal detectors with a time-limited integration window.

It is worth noting the ingenious techniques used by Bloch to control stimulus duration and luminance in the absence of oscilloscopes, computers, and modern photometers. A pair of well-calibrated slits on the opposite ends of a blackened cardboard box spinning at a constant angular speed allowed millisecond control of duration, the regime within which his law operates. For any given duration, Bloch expressed the visibility threshold of a candle he placed 0.1 m behind a translucent white sheet of paper in terms of the distance of another candle placed X m further away and yielding the same visibility in the absence of the paper screen as well as (though indirectly, presumably relying on the inverse square law) in terms of the distance of such candle placed this time in front of the white paper so that the latter appear equally bright as when lit from behind.

### Recent Developments and Outlook

All studies above focused on sensory *detection* for which the transfer function and Bloch’s curve are—given the linear systems approach—interchangeable empirical descriptors (once the impulse response of the system is known). Nonetheless, starting perhaps with Ratcliff (1978), drift diffusion became the standard modeling approach to information accumulation over time for both threshold and suprathreshold stimuli and thereby to its relation with response time (e.g., Bogacz, Brown, Moehlis, Holmes, & Cohen, 2006; Brunton, Botvinick, & Brody, 2013; Gold & Shadlen, 2001, 2007; Usher & McClelland, 2001).

Drift-diffusion (also referred to as bound diffusion or integration-to-bound) models were and still are meant to account for subjects’ decision time and, critically, for its stochastic variability over time in the presence of an ongoing stimulation. In principle, they can also account for the stimulation intensity required to reach a decision as a function of stimulus duration (Bloch’s curve). Equivalently, the linear systems approach used to account for Bloch’s curve could also be enhanced to account for the decision time and its variability. The possibility of such enhancement has been only rarely pointed out (Gorea, Belkoura, & Solomon, 2014; Smith, 1998; Smith & Ratcliff, 2009). Based on their measures of the temporal transfer function and of Bloch’s curve for the extraction of the average size of a number of variable-size visual items, Gorea et al. (2014) inferred that the temporal impulse response underlying average size extraction is a delta function, that is an instantaneous operator (as long as the items are suprathreshold).

The use of an enhanced linear systems approach for the characterization of the temporal properties of higher order processes remains a valuable alternative to current drift-diffusion models.

### The Highlighted Paper

As information accumulation for a wide range of perceptual tasks remains a hot research topic, it is worth offering the public the original work of Adolphe-Moïse Bloch in its first and only English translation.

## Experiments in Vision

Bloch, A.-M. (1885) Expériences sur la vision [Experiments in vision]. *Comptes Rendus de Séances de la Société de Biologie, Paris 37*(28), 493–495. (Translation by A. Gorea with illustrations and notes)

The present experiments mainly address the following question: Is it possible to present a luminous object sufficiently briefly so that it will not be seen?

A certain number of physiologists have studied this problem, and, most recently, Mr. Richet and Mr. Breguet concluded affirmatively. They designed an ingenious device allowing fast and regular eclipses of a light presented in front of the observer; the visual excitation time was, according to these authors, of about 1/1000 seconds; under such conditions, the luminous body, partly attenuated by a smoked glass plate became invisible.

I thought that this result that can be regarded as *quantitative* is not entirely satisfying, and that it would be interesting to specify precisely the duration as well as the intensity of the visual excitation.

To this end, I used a Foucault regulator.^1^ I placed on the vertical axis that bears the blades a blackened cardboard box with two slots carved into two opposite faces and placed a candle at a specified distance in front of the box.

I observed the light through the end of a copper, meter-long tube whose end had been filled with wax and pierced with a half-millimeter diameter aperture.

As the motor was running, I recorded the instrument’s vibrations on the blackened cardboard: double vibrations of 1/250 seconds.

I could hence precisely assess the time for one slot to pass in front of the tube’s aperture and, as a consequence, the duration of the visual excitation produced by the candle for each turn of the motor. Skipping the computational details, I would say that for a slot of 0.5 mm, the duration was 1/1119 seconds.

Under these conditions, the candle becomes invisible when a sheet of translucent paper is placed between the candle and the spinning box at 0.09 to 0.10 m from the former.

I subsequently compared, by means of standard photometric procedures, the intensity of this candle behind the screen and that of an isolated candle and found that the latter must be set at a distance of 1.65 m from a Bouguier photometer^2^ to yield an equivalent lighting. The experiment can thus be summarized as follows:

A luminous body of equal intensity to a white paper lit from behind by a candle 1.65 m away becomes invisible when it is displayed for 1/1119 seconds.

However, the translucence of the paper varies with its thickness. It was therefore necessary to compare the lighting showing through this paper with the lighting reflected by a white surface lit by a candle placed in front of rather than behind it. The photometric measurement showed that the reflected light was 4.5 times more luminous. It can thus be concluded that a white sheet of paper directly lit for 1/1119 seconds by a candle 3.47 m away becomes invisible.

This first experiment naturally raised a number of problems.

1. If the duration of the light varies, how should its intensity vary such that visual perception still has no time to develop?
2. Is there a law describing the relationship between the duration of a light and its perceived intensity?
3. Can these experiments be applied to new photometric measures?

These are questions that I can only raise today and that I’ll bring up in future studies. I shall only mention the following experiments:

The aperture of the tube being equal to the area of a 0.0025 m side square, when the slots in the box are 0.0005, 0.001, 0.0015, 0.0025, 0.007, and 0.010 m,^3^ the lighting duration varies from 0.00173 to 0.0518 seconds^4^; when the distance of the screen, mounted on a cart in front of the candle, is varied, *the [visible] light is markedly in inverse proportion to its duration*. This is to say that, in order to obtain the cessation of visual sensation, doubling the intensity of the light requires halving its duration. These results are subject to pending verifications as they are not as yet sufficiently substantiated. They demonstrate nonetheless their putative practical use in photometry. This procedure should allow the measurement of light that current methods cannot assess as they are by construction comparative while my method is direct.

As of the accuracy of my method, I believe it to be comparable to that of current photometers; suffice to mention that sun’s light compared with that of the moon is estimated to be 300 times larger by some measurement devices and 800 times larger by others, a range that demonstrates the poor precision of the scientific photometric experiments currently carried out.

## Notes

1 The “Foucault regulator” refers to a double conical pendulum whose rotation speed modulates the gap between its rotating spheres or blades. Foucault’s goal was to increase the sensitivity of the device so that a minute speed change entails a large gap-change between the balls or blades. To do so, he used a system of counterweights or of springs (Figure 1). *All notes are by the translator*.

2 Pierre Buguer (misspelled by Bloch; 1698–1758) was a French mathematician, geophysicist, geodesist, astronomer, founder of photometry, and one of the founders of physiological optics, also known as “the father of naval architecture” (see Morère, 1965). His very simple photometer is illustrated in Figure 2.

3 The units are not specified but, given the context, they must be meters. The slits should therefore range from 0.5 to 10 mm.

4 The most cited temporal integration constant is 50 ms (but see Gorea & Tyler, 1986).
